# Compound C enhances the anticancer effect of aspirin in HER-2-positive breast cancer by regulating lipid metabolism in an AMPK-independent pathway

**DOI:** 10.7150/ijbs.39936

**Published:** 2020-01-01

**Authors:** Ying Wu, Bohua Yan, Wenqin Xu, Lili Guo, Zhe Wang, Guoyin Li, Niuniu Hou, Jian Zhang, Rui Ling

**Affiliations:** 1 Department of Thyroid, Breast and Vascular Surgery, Xijing Hospital, Fourth Military Medical University, Xi'an, Shaanxi, China.; 2 Key Laboratory of Cancer Biology, Department of Biochemistry and Molecular Biology, Fourth Military Medical University, Xi'an, Shaanxi, China.; 3 Fourth Military Medical University, Xi'an, Shaanxi, China.; 4 Department of Ophthalmology, Eye Institute of China PLA, Xijing Hospital, Fourth Military Medical University, Xi'an, Shaanxi, China.; 5 College of Life Science and Agronomy, Zhoukou Normal University, Zhoukou, Henan, China.

**Keywords:** HER-2-positive breast cancer, aspirin, Compound C, lipid metabolism, c-myc.

## Abstract

Various clinical studies have determined that aspirin shows anticancer effects in many human malignant cancers, including human epidermal growth factor receptor-2 (HER-2)-positive breast cancer. However, the anti-tumor mechanism of aspirin has not been fully defined. The aim of this study was to determine the role of Compound C in enhancing the anticancer effect of aspirin. HER-2-positive breast cancer cell lines were treated with aspirin with or without Compound C pre-treatment; their phenotypes and mechanisms were then analyzed* in vitro* and *in vivo*. Aspirin exhibited anticancer effects in HER-2-positive breast cancer by inhibiting cell growth and inducing apoptosis through the activation of AMP-activated protein kinase (AMPK). Unexpectedly, pre-treatment with Compound C, a widely used AMPK inhibitor, induced robust anticancer effects in cells compared to aspirin monotherapy. This anticancer effect was not distinct in HER-2 negative breast cancer MDA-MB-231 cells and may be due to the inhibition of lipid metabolism mediated by c-myc. Besides, c-myc re-expression or palmitic acid supply could partially restored cell proliferation. Aspirin exhibits anticancer effects in HER-2-positive breast cancer by regulating lipid metabolism mediated by c-myc, and Compound C strengthens these effects in an AMPK-independent manner. Our results potentially provide a novel therapeutic strategy exploiting combined aspirin and Compound C therapy for HER-2-positive breast cancer, which acts by reducing *de novo* lipid synthesis.

## Introduction

Breast cancer is the most common type of malignant tumor in women worldwide, and is the second leading cause of cancer-related death in females globally 1. Human epidermal growth factor receptor-2 (HER-2) is found in approximately 30% of breast cancer patients 2. It plays important roles in promoting breast cancer invasion and metastasis 3, and acts as an important biomarker for diagnosis and as a target for the clinical treatment of breast cancer patients. Although there are many drugs targeting HER-2-positive breast cancer, such as the humanized monoclonal antibody trastuzumab and the HER-2 kinase inhibitor lapatinib, poor prognosis and high drug resistance still threaten the lives of HER-2-positive breast cancer patients 4. Thus, the discovery of new and more effective drugs for HER-2-positive breast cancer treatment is of great urgency and necessity.

Aspirin has been reported to function as a potential chemo-preventive drug for several types of cancer, including colorectal, stomach, esophageal, lung 5, prostate cancer 6, and hepatocellular carcinoma 7. Daily aspirin intake (≥ 75 mg) was observed to reduce the risk of distant metastasis in subsequent follow-ups of colorectal cancer patients without initial metastasis 5. Aspirin is known to inhibit cancer cell growth and induce cancer cell apoptosis both *in vitro* and *in vivo* 8, 9 by inhibiting cyclooxygenase (COX) enzyme activity, which leads to the suppression of endogenous prostaglandins (PG) including PGE2, PGD2, and thromboxane A2 (TXA2); these factors are known to trigger pain, inflammation, fever, or blood clotting. The antitumor effect of aspirin is partially due to the inhibition of COX and the activation of platelet production 10. Activated platelets not only stimulate the expression of COX2 in epithelial cells, which has diverse effects on cell proliferation and survival, but also improve the spreading of cancer cells 10. Aspirin can directly activate AMP-activated protein kinase (AMPK) signaling 11, which is a key regulator of cellular metabolism and energy homeostasis in mammalian tissues; AMPK is known to be central for the regulation of several major signaling pathways 12. After oral administration of aspirin, it is absorbed through the gastrointestinal system and converted into salicylic acid; free salicylic acid can directly bind AMPK to activate it 13. Moreover, aspirin may also indirectly activate AMPK through other processes 14. It has been reported that aspirin and its metabolites regulate cyclin-dependent kinase (CDK) 15, 16 and Wnt/β-catenin signaling 17, 18.

In this study, we used Compound C, a widely used AMPK inhibitor, to examine whether the inhibition of AMPK would affect the anticancer activity of aspirin. Surprisingly, pre-treatment with Compound C enhanced the aspirin-induced growth inhibition effect in HER-2-positive breast cancer cells, despite blocking AMPK activity. At the same time, we found that Compound C pre-treatment reduced the expression of c-myc compared to cells treated with aspirin only; thus, we concluded that Compound C intensified the anticancer effect of aspirin independent of AMPK activity, and possibly through some other mechanism.

It is well known that cancer cell proliferation is typically accompanied by *de novo* lipogenesis, which plays a structural role in the construction of new cell membranes and is also likely to be further involved in signaling events that are important for cancer development 19, 20. In this study, we observed that combined Compound C and aspirin treatment inhibited fatty acid synthesis and the expression of key enzymes in *de novo* lipogenesis, such as sterol regulatory element-binding protein 1 (SREBP1), stearoyl-CoA desaturase 1 (SCD1), fatty acid synthase (FASN), and ATP citrate lyase (ACLY).

Restoring the expression of c-myc in treated-HER-2-positive breast cancer cells restored fatty acid synthesis and cell viability to some extent. Our *in vivo* mouse model also verified that Compound C intensified the anti-tumor effect of aspirin and further inhibited SREBP1/SCD1 expression that was down-regulated by aspirin in HER-2-positive breast xenograft tumors. Thus, our results showed that Compound C enhanced the inhibitory effect of aspirin by inhibiting *de novo* lipogenesis regulated by c-myc in an AMPK-independent manner.

## Materials and methods

### Cell lines and cell culture

The human HER-2-positive breast cancer cell lines AU-565, BT-474, and SKBR-3 were obtained from the American Type Culture Collection (ATCC, Manassas, VA, USA). The AU-565 and SKBR-3 cell lines were cultivated in Roswell Park Memorial Institute (RPMI) 1640 medium (Gibco, Waltham, MA, USA), while BT-474 cells were cultured in Dulbecco's Modified Eagle's Medium (DMEM; Gibco). Both RPIM 1640 and DMEM were supplemented with 10% fetal bovine serum (FBS) and penicillin (100 U/ml) and streptomycin (100 µg/ml; Beyotime, Jiangsu, China). All cells were maintained in a 37 °C humidified incubator with 5% CO_2_.

### MTT assay

Cell proliferation was measured by a 3-(4, 5-dimethylthiazol-2-yl)-2, 5-diphenyltetrazolium bromide (MTT) assay (Sigma Aldrich, St. Louis, MO, USA). MTT was dissolved in phosphate-buffered saline (PBS). Cells in the exponential growth period were seeded in 96-well cell culture plates (Thermo Fisher Scientific, Waltham, MA, USA) in a final volume of 200 μL with 8.0 × 10^3^ cells per well. After attachment for 24 h, cells were treated with different doses of aspirin (dissolved in dimethyl sulfoxide [DMSO]; Sigma Aldrich) with or without Compound C (dissolved in DMSO; Selleck Chemicals, Houston, TX, USA) pre-treatment (control cells were instead incubated with an equal volume of DMSO) in 200 μL of the relevant medium. After the indicated time period, 5% MTT was added to each well for an additional 4 h. Formazan crystals were solubilized in each well in 150 μL DMSO after the removal of MTT. The absorbance in each well at 490 nm was measured using a spectrophotometer (Bio-Rad Laboratories, Hercules, CA, USA). Five wells were used for each aspirin dose in three independent experiments.

### Flow cytometry for apoptosis and cell cycle analysis

Cells were cultured in FBS-depleted medium for 8 h and treated with aspirin for 24 h with or without Compound C pre-treatment. For apoptosis analysis, dead and apoptotic cells were collected, washed with ice-cold PBS, measured with an Annexin V-PE Apoptosis Detection Kit (Oncogene Research Products, La Jolla, CA, USA) according to the manufacturer's instructions, and then analyzed by flow cytometry (Becton Dickinson, Franklin Lakes, NJ, USA). For the cell cycle assay, cells were treated with 0.05% trypsin-ethylenediaminetetraacetic acid (EDTA) and washed with ice-cold PBS; resuspended cells were fixed with 1 ml cold 70% ethanol and then incubated at 4 °C for at least 4 h. Fixed cells were rehydrated in flow cytometry buffer and subjected to propidium iodide (PI)/RNase staining. Cells in different cell cycle phases were detected by flow cytometry using a FACScan instrument (Becton Dickinson), and analyzed with CellQuest software (Becton Dickinson). All experiments were repeated three times independently.

### Western blotting

Cells were plated on 60-mm plates, grown to 70% confluency, and treated with aspirin with or without Compound C pre-treatment, as described above. Then, the cells were harvested and washed with ice-cold PBS, and then lysed in radio-immunoprecipitation assay (RIPA) lysis buffer (100 mmol/L NaCl, 50 mmol/L Tris-HCl pH 7.5, 1% Triton X-100, 1 mmol/L EDTA, 10 mmol/L β-glycerophosphate, 2 mmol/L sodium vanadate, and 1 mmol/L protease inhibitor). The protein concentration was assessed using the micro-bicinchoninic acid (BCA) protein assay kit (Thermo Fisher Scientific). Samples containing 60 μg total proteins were separated by 12% or 15% SDS-PAGE and transferred to polyvinylidene fluoride (PVDF) membranes (Merck Millipore, Burlington, MA, USA). Membranes with immunoreactive bands were blocked with 5% non-fat dry milk in 0.01% Tris-buffered saline (TBS)/Tween 20 (TBST) for 2 h, followed by incubation with primary antibodies targeting β-actin, AMPKα, p-AMPKα, β-catenin, c-myc (Cell Signaling Technology, Danvers, MA, USA), FACN, and ACLY (Abcam, Cambridge, UK), SREBP1 and SCD1 (Proteintech, Wuhan, China) diluted in TBST according to the suppliers' recommendations at 4 °C overnight. After washing three times with TBST for 15 min each, the membranes were incubated with horseradish peroxidase (HRP)-labeled secondary antibodies (1:2000; Cell Signaling Technology) in TBST at room temperature for 1 h. After an additional four washes with TBST, membranes with immunoreactive bands were exposed on a Kodak X-Omat processor (Rochester, NY, USA).

### Immunofluorescence staining

Cells were seeded in confocal dishes (Thermo Fisher Scientific), grown to 30% confluence, and treated with aspirin for 24 h with or without Compound C pre-treatment for 4 h. The media was removed, and the cells were fixed in 4% paraformaldehyde for 15 min at room temperature. The cells were washed with PBS three times for 5 min each, and then permeabilized in 0.5% Triton X-100 at 37 °C for 15 min. After washing a further three times in PBS for 5 min each, primary antibodies targeting c-myc (1:200; Santa Cruz Biotechnology, Houston, TX, USA) and β-catenin (1:250) were diluted in PBS, added to the cells, and incubated at 4 °C overnight. Thereafter, the cells were washed three times in PBS for 5 min each, and incubated with secondary CY3-conjugate anti-mouse IgG antibodies (Sigma Aldrich) at a dilution of 1:100 for 2 h at room temperature in the dark. After washing again, the cells were incubated with DAPI (Sigma Aldrich; 5 μg/ml) in the dark for 5 min to stain the nuclei. All images were captured using an Axiovision camera with a Carl Zeiss zoom inverted florescence microscope (Carl Zeiss, Oberkochen, Germany) at a magnification of 60 ×.

### Colony formation assay

Cells in the exponential growth period were seeded in 6-well plates in triplicate at a density of 2.0 × 10^3^ cells per well, and treated with 2.5 mmol/L aspirin with or without Compound C pre-treatment after attachment. After 15 d, the media was removed, and the colonies were washed twice with PBS. The cells were fixed in methanol (Sigma Aldrich) for 15 min and stained with Giemsa (Sigma Aldrich) for 20 min. Colonies containing more than 50 cells were counted.

### Liquid chromatography-tandem mass spectrometry

For the following experiments, 1 × 10^6^ cells were washed in cold PBS, trypsinized, and collected; cell pellets were frozen at -80 °C. An aliquot of 500 μL carbinol was added to each extract. The sample extracts were centrifuged at 4 °C and 8,000 × *g* for 15 min.

The supernatant was placed in a polypropylene microtiter plate. Methanolic internal standard solution (150 μL) was then added manually. The microtiter plate was gently shaken for 30 min to extract the acylcarnitine markers. The methanol extract was then manually transferred to a second polypropylene microtiter plate and dried using a Pressure Blowing Concentrator (Cence, Hunan, China) (50 °C). Butanol-HCl (60 μL) was manually placed in each sample well, and the microtiter plate was covered with a thin Teflon sheet under a heavy weight and placed in a forced air oven at 70 °C for 15 min. After the plate was removed from the oven, the Pressure Blowing Concentrator was used to remove the butanol-HCl. The butanol-derivatized samples were reconstituted with 100 μL acetonitrile (Sigma Aldrich) and distilled water (70:30, v/v) and each plate was covered with aluminum foil. The samples were then subjected to liquid chromatography-tandem mass spectrometry (LC-MS/MS) analysis.

### Liquid chromatography

Samples were analyzed using LC-MS/MS (Shimadzu-LC20AD, Kyoto, Japan; AB-Sciex API 4000+, Framingham, MA, USA). A mixture of acetonitrile and distilled water (70:30, v/v) was used as the carrier solution at a flow rate of 0.14 ml/min. A step gradient program was developed for the optimal separation of amino acids: 0.14 ml/min for 0.2 min, 0.03 ml/min for 1 min, and 0.3 ml/min for 0.2 min. Next, 18 μl sample was injected into the carrier solution, and the stream was introduced to the ion source for MS/MS without column separation.

### Mass Spectrometry

The MS parameters in positive ion mode (ESI + MODE) were as follows: heater temperature, 300 °C; capillary temperature, 350 °C; sheath gas flow rate, 45 arb; aux gas flow rate, 15 arb; sweep gas flow rate, 1 arb; spray voltage, 3.0 kV. The scan function for free carnitine (35 V, 17 eV) was used to determine the ion abundance of the fragment ions at 103 m/z, and acylcarnitines (35-54 V, 25 eV) were used to determine the ion abundance of the fragment ions at 85 m/z. All data acquisition and processing were carried out with Analyst 1.5.2 software (AB-Sciex).

### Oil red O staining

Cells were seeded in 6-well plate in triplicate and treated with 5 mmol/L aspirin with or without Compound C pre-treatment. After 24 h, the culture media was removed and cells were washed three times with PBS. Next, the cells were fixed with 4% paraformaldehyde at room temperature for 30 min, and rinsed with PBS once for 1 min. Thereafter, the cells were washed with 60% isopropanol for 15 s. Freshly diluted Oil Red O working solution (Hat Biotechnology, Xian, China) was then added, and the cells were incubated for 60 min at room temperature. Then, cells were rinsed again with 60% isopropanol for 15 s and washed three times with PBS for 5 min each. All images were captured using an Axiovision camera with a Carl Zeiss zoom inverted florescence microscope at a magnification of 60 ×. For the quantitation of lipid loading, the cells were seeded in 96-well plates and treated as described above. After removing the staining solution, the dye retained in the cells was eluted with isopropanol and the optical density at 540 nm (OD_540_) was determined with a spectrophotometer (Bio-Rad Laboratories).

### Plasmid and gene transfection

Cells were seeded in 60-mm plates and grown to 60% confluence. Expression vectors for pcDNA3.1-c-myc or empty vector were transfected into cells using Lipofectamine 2000 (Thermo Fisher Scientific) according to the manufacturer's instructions. The final concentration of pcDNA3.1-c-myc and empty vector was 8 μg per plate. After 24 h, cells were treated with aspirin with or without pre-treatment with Compound C. After another 24 h, the cells were harvested for western blotting analysis as described above.

### RNA isolation and quantitative PCR

Total RNA was extracted from breast cancer cells using TRIzol reagent (Invitrogen, Carlsbad, CA, USA) according to the manufacturer's instructions. cDNA was synthesized using the PrimeScript RT reagent Kit (Takara Bio, Shiga, Japan) in a total volume of 20 μl according to the manufacturer's instructions. PCR was performed with target-specific primers using the Bio-Rad 7900 Sequence Detection System and the SYBR Green qPCR system (Takara) according to the manufacturer's instructions; β-actin was used as an internal control. Briefly, reactions were performed in triplicate containing 5 μl 2× SYBR Premix II, 2 μl cDNA (corresponding to 50 ng RNA/reaction), and 1 μl 10 mM primers in a final volume of 10 μl. All data were analyzed using the 2^-ΔΔCT^ method, and mRNA levels were normalized to β-actin. The following primers were used:

β-actin: 5′-AAGAGAGGCATCCTCACCCT-3′

and 5′-TACATGGCTGGGGTGTTGAA-3′.

SREBP1: 5′- ACAGTGACTTCCCTGGCCTAT-3′

and 5′-GCATGGACGGGTACATCTTCAA-3′.

SCD1: 5′-TCTAGCTCCTATACCACCACCA-3′

and 5′-TCGTCTCCAACTTATCTCCTCC-3′

FASN: 5′-AAGGACCTGTCTAGGTTTGATGC-3′

and 5′-TGGCTTCATAGGTGACTTCCA-3′

ACLY: 5′-TCGGCCAAGGCAATTTCAGAG-3′

and 5′-CGAGCATACTTGAACCGATTCT -3′

### Animals and ethical permission

For animal studies, 4-6-week-old female nude mice (approximately 20 g) were obtained from the Experimental Animal Center at the Fourth Military Medical University (Xian, China). A total of 2 × 10^6^ SKBR-3-luciferase-positive cells were suspended in sterile PBS and injected subcutaneously into the right flank of each mouse. After 1 week, when tumors reached approximately 50-100 mm^3^, the mice were randomly divided into three groups (n = 6 per group): (a) control (DMSO dissolved in 200 μl corn oil administered once daily by intraperitoneal injection); (b) aspirin administration by gavage (100 mg/kg, once daily); (c) Compound C (0.2 mg/kg, once daily by intraperitoneal injection) and aspirin (100 mg/kg, once daily by intragastric injection). The tumors were closely monitored with a whole-body fluorescent imaging system every 3 d by injecting 200 μL luciferin (15 mg/ml) intraperitoneally and measuring the bioluminescence signal for 60 s; body weights were measured to monitor the well-being of the animals. The mice were closely monitored for 30 days and then sacrificed to remove the tumors for subsequent immunohistochemistry (IHC) assays. Mice were maintained at an artificial 12/12 h light/dark regime with access to food and water *ad libitum*. All protocols were approved by the Institutional Animal Care and Use Committee of Fourth Military Medical University and complied with institutional guidelines.

### Immunohistochemistry

Human SREBP1 rabbit polyclonal antibody and human SCD1 rabbit polyclonal antibody (Proteintech, Wuhan, China) were used to detect protein on formalin-fixed, paraffin-embedded (FFPE) sections from nude mice in this experiment. Tissues were sliced into 5-μm-thick sections with a microtome, transferred onto adhesive slides, and then dried at 62 °C for 30 min. All slides were deparaffinized and rehydrated using xylene and graded ethanol. After deparaffinization and antigen retrieval for 30 min in citrate buffer (pH 6.0), the slides were blocked with 5% bovine serum albumin for 1 h and incubated overnight at 4 °C in primary antibody (anti-SREBP1, 1:100; anti-SCD1, 1:100). Subsequently, sections were washed with PBS three times for 5 min each and labeled with goat anti-rabbit HRP-conjugated secondary antibodies for 20 min at 37 °C.

The positively stained area was scored on a scale of 0-4: 0, no staining; 1, poor; 2, moderate; 3, moderate to strong; 4, strong. The frequency of positive cells was defined as follows: 0, less than 5%; 1, 5-25%; 2, 26-50%; 3, 51-75%; and 4, greater than 75%. When staining was heterogeneous, it was scored as follows: each component was scored independently and summed for the results. For example, a specimen containing 75% tumor cells with moderate intensity (3 × 2 = 6) and another 25% tumor cells with weak intensity (1 × 1 = 1) received a final score of 6 + 1 = 7. For statistical analysis, scores of 0-7 were considered as low expression and scores of 8-12 were considered as high expression.

### Statistical analysis

All experiments were repeated three times independently and all data were given as the mean ± standard deviation. The significance between different groups was evaluated using Student's* t*-test, χ^2^ test, or one-way analysis of variance (ANOVA) using SPSS 19.0 software (SPSS, Chicago, IL, USA). A value of *p* < 0.05 was considered statistically significant.

## Results

### Aspirin induces growth suppression in HER-2-positive breast cancer cells

**Figure 1:**
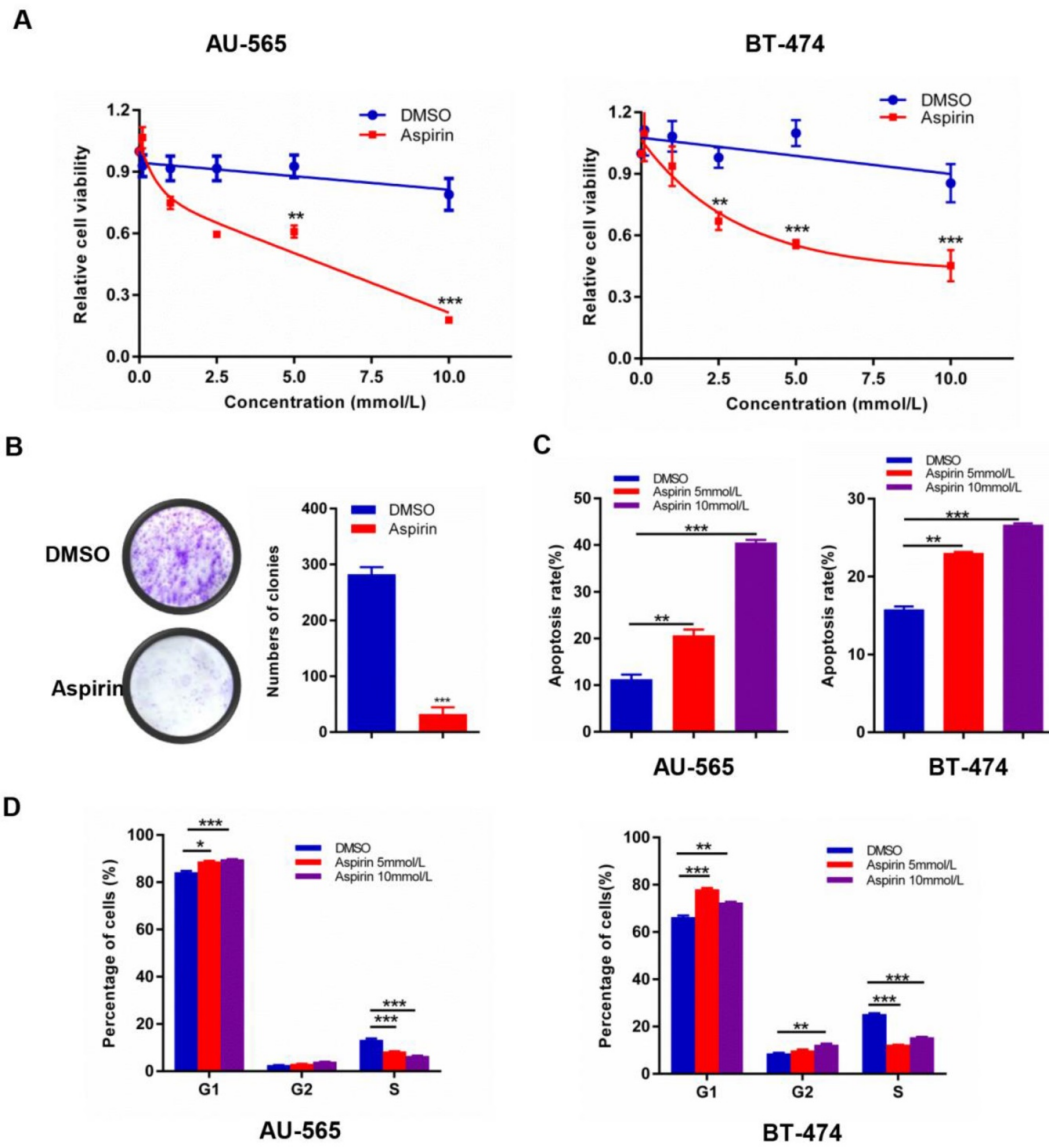
Aspirin exerts anticancer activity in HER-2-positive breast cancer cells. **A.** AU-565 and BT-474 were treated with various concentrations of aspirin and equal volumes of DMSO as a control for 24 h; cell viability was determined by an MTT assay. **B.** Colony formation of AU-565 cells was inhibited by aspirin. **C, D** Flow cytometry showed that aspirin induced cell apoptosis (**C**) and cell cycle arrest (**D**) in HER-2-positive breast cancer AU-565 and BT-474 cells. *P < 0.05; **P < 0.01; ***P < 0.001 vs. control.

Experimental investigation of the growth suppression effects of aspirin on HER-2-positive breast cancer cells revealed that aspirin could effectively inhibit human HER-2-positive breast cancer cell viability in a dose-dependent manner; the IC_50_ values for AU-565 and BT-474 were 5 mM (Figure 1A). Furthermore, aspirin inhibited the colony-forming ability of AU-565 cells (Figure 1B). As shown in Figure 1 C-D, aspirin enhanced the apoptosis rates of AU-565 and BT-474 cells in a dose-dependent manner, as determined by flow cytometry. Similarly, as shown in the MTT assay, aspirin reduced the proportion of S-phase cells in AU-565 and BT-474 cell lines, as well as increased the proportions of G1- or G2/M-phase cells at the same time point. These results revealed the anti-tumor effect of aspirin on HER-2-positive breast cancer cells.

### Compound C enhances the anticancer effect of aspirin on HER-2-positive breast cancer cells

**Figure 2:**
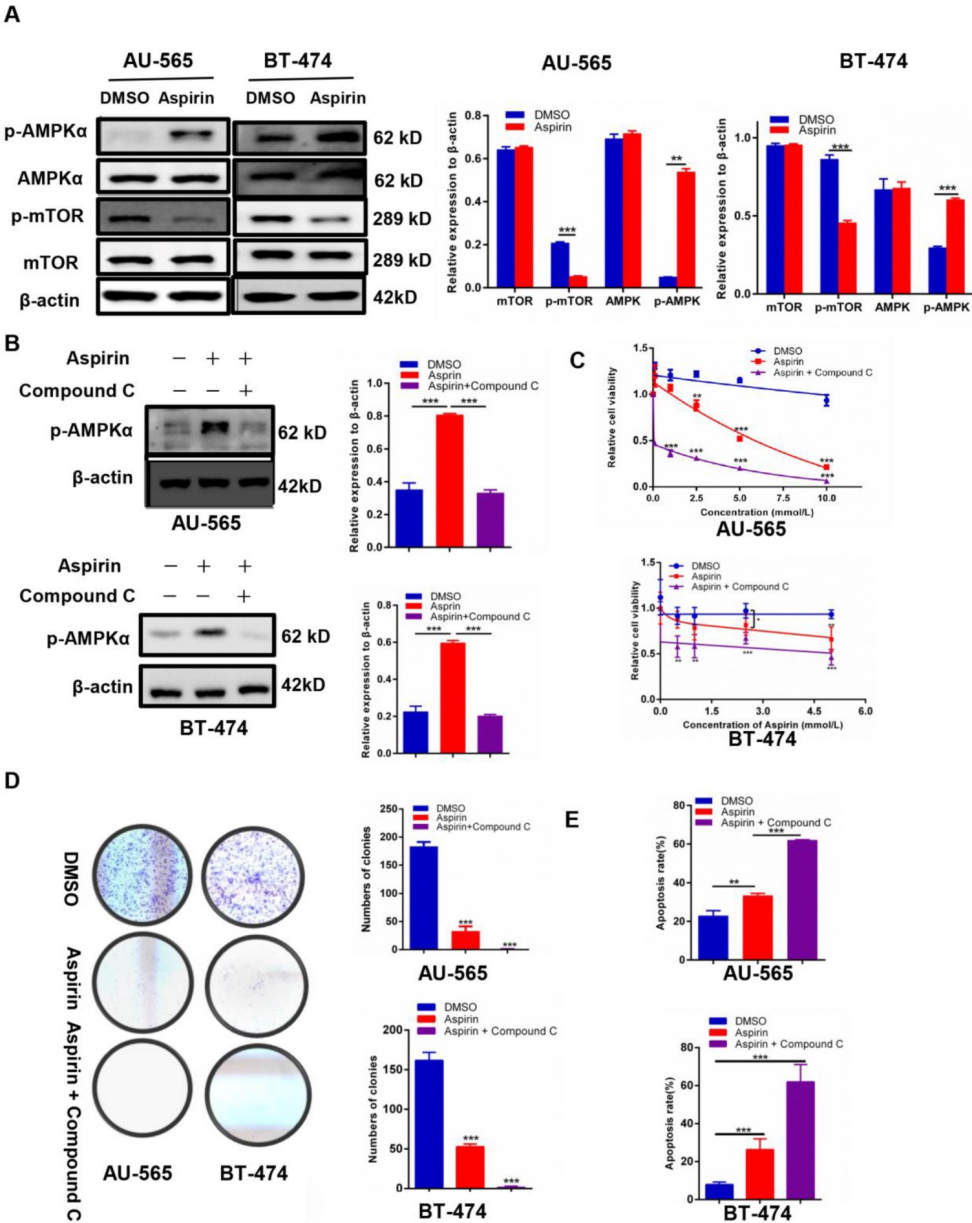
Compound C enhances the anticancer effect of aspirin in HER-2-positive breast cancer cells. **A.** Western blotting showed that aspirin can activate AMPKα phosphorylation and inhibit p-mTOR expression in AU-565 (left) and BT-474 (right) cells. **B.** Compound C successfully inhibited the expression of p-AMPK elevated by aspirin in AU-565 (top) and BT-474 (bottom) cells. **C.** An MTT assay showed that Compound C (10 μmol/L, 4 h), combined with aspirin, reduced cell viability to a greater extent in AU-565 (top) and BT-474 (bottom) cells. **D.** Compound C enhanced the aspirin-induced inhibition of AU-565 and BT474 cell colony formation ability. **E.** Flow cytometry showed that Compound C promoted aspirin-induced apoptosis in AU-565 and BT-474 breast cancer cells. *P < 0.05; **P < 0.01; ***P < 0.001 vs. control.

We found that p-AMPKα was elevated and p-mTOR was inhibited in aspirin-treated HER-2-positive breast cancer cells (Figure 2A). Because Compound C has been extensively used as an AMPK inhibitor 21, we measured whether Compound C-induced AMPK inhibition would rescue the anti-growth effect of aspirin on HER-2-positive breast cancer cells. Western blotting showed that Compound C inhibited the expression of aspirin-activated p-AMPKα in AU-565 and BT-474 breast cancer cells (Figure 2B) and HER-2-negative breast cancer MDA-MB-231 breast cancer cells (Supplementary Figure 1A). The MTT assay showed that HER-2-positive breast cancer cells pre-treated with Compound C increased the effect of aspirin in terms of cell growth inhibition, which was highly unexpected (Figure 2C), although the result in MDA-MB-231 breast cancer cells was not significant with Compound C pretreatment (Supplementary Figure 1B). The colony formation assay showed a significant decrease in the colony numbers of Compound C-pre-treated AU-565 and BT-474 cells compared to those of aspirin alone-treated cells (Figure 2D). Through flow cytometry, Compound C was found to increase aspirin-induced apoptosis in HER-2-positive breast cancer cells (Figure 2E), but not in HER-2-negative breast cancer MDA-MB-231 cells (Supplementary Figure 1C). These results strongly indicated that Compound C did not reverse the anti-growth effect of aspirin on HER-2-positive breast cancer cells; in contrast, it strengthened the anti-proliferation and pro-apoptosis effects of aspirin in an alternative and AMPK-independent manner in HER-2-positive breast cancer cells.

### Compound C strengthens the aspirin-induced attenuation of lipid metabolism in HER-2-positive breast cancer cells

**Figure 3:**
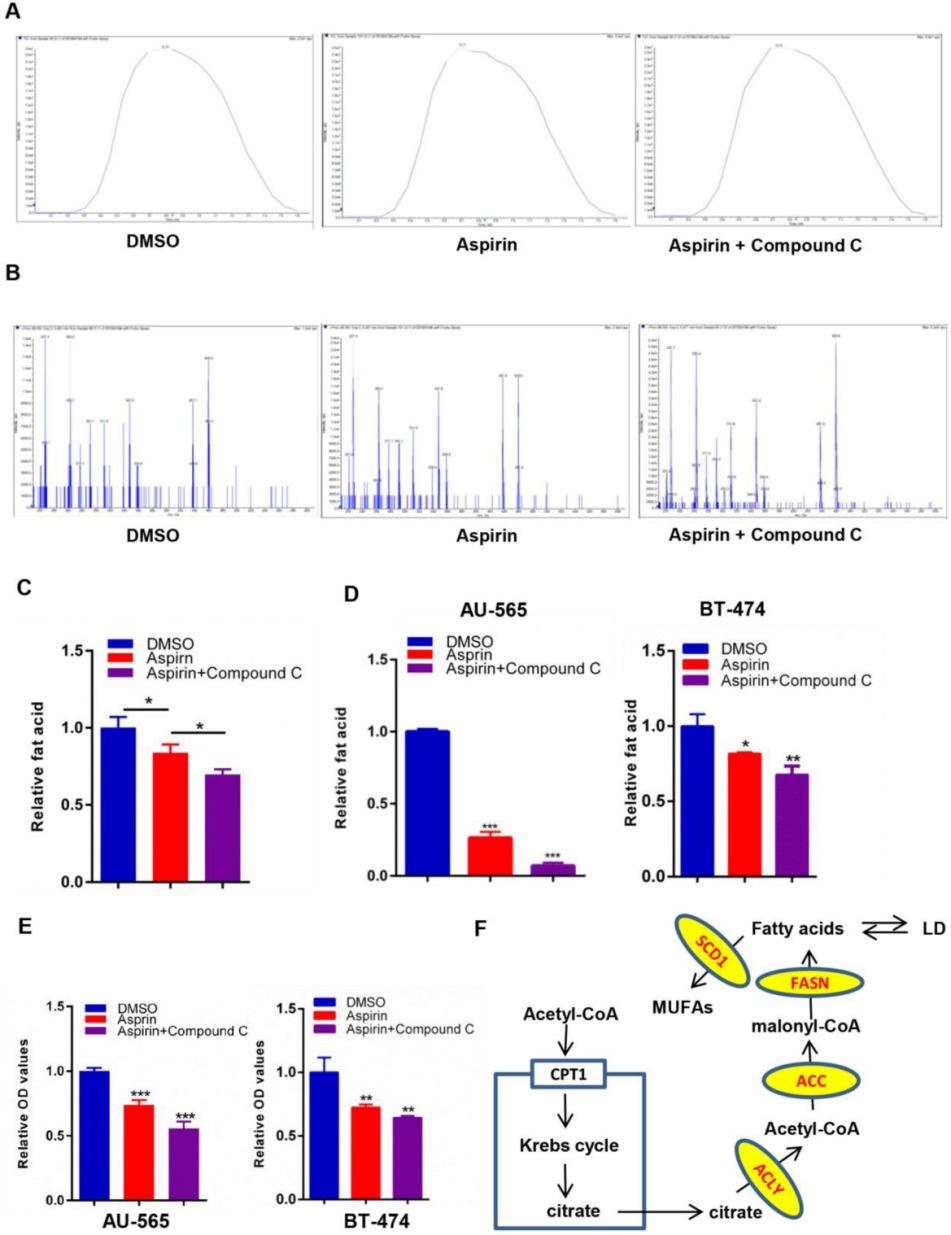
Compound C exacerbates aspirin-induced lipogenesis inhibition. **A, B.** LC-MS/MS was performed to investigate the levels of fatty acid production in 3 groups of SKBR-3 breast cancer cells: DMSO as control, Aspirin alone, Aspirin and Compound C. **A.** Total ions chromatograph of the 3 groups of SKBR-3 breast cancer cells as above. **B.** Precursor ion scans for 3 groups of SKBR-3 breast cancer cells as above. **C.** Quantification of lipid LC-MS/MS and Compound C enhanced aspirin-impaired lipogenesis in SKBR-3 breast cancer cells. **D. E.** An Oil red O staining assay showed that Compound C exacerbated aspirin-impaired lipogenesis in AU-565 and BT-474 breast cancer cells. **F.** Lipid metabolism in breast cancer cells.

Growing evidence suggests that aspirin exerts antitumor activity associated with reduced *de novo* fatty acid synthesis, which is a common feature of cancer to meet the biosynthetic demands of a growing tumor. To investigate changes in the levels of lipogenesis in HER-2-positive breast cancer cells after aspirin treatment with or without Compound C pre-treatment, we performed a LC-MS/MS assay. We found that aspirin attenuated the levels of lipid formation. Strikingly, we confirmed that Compound C pre-treatment decreased lipid formation in SKBR-3 cells to a greater extent compared to that in the aspirin-treated group (Figure 3A-C). Moreover, Oil red O staining showed that aspirin treatment reduced the production of lipids in AU-565 and BT-474 cells, and that Compound C pre-treatment enhanced this effect in HER-2-positive breast cancer cells (Figure 3D-E). But in HER-2-negative breast cancer MDA-MB-231 cells, the difference in Compound C pre-treated group and aspirin-alone treated group was not significant (Supplementary Figure 1D-E), and we found distinct lipid droplets distribution and cellular morphological changes. Thus, our data demonstrated broad changes in the lipid metabolism in aspirin-treated HER-2-positive breast cancer cells with or without Compound C pre-treatment, and that Compound C might be a novel inhibitor of lipid metabolism in HER-2-positive breast cancer cells in combination with aspirin.

### Aspirin and Compound C down-regulate key markers of lipogenesis in HER-2-positive breast cancer cells

**Figure 4:**
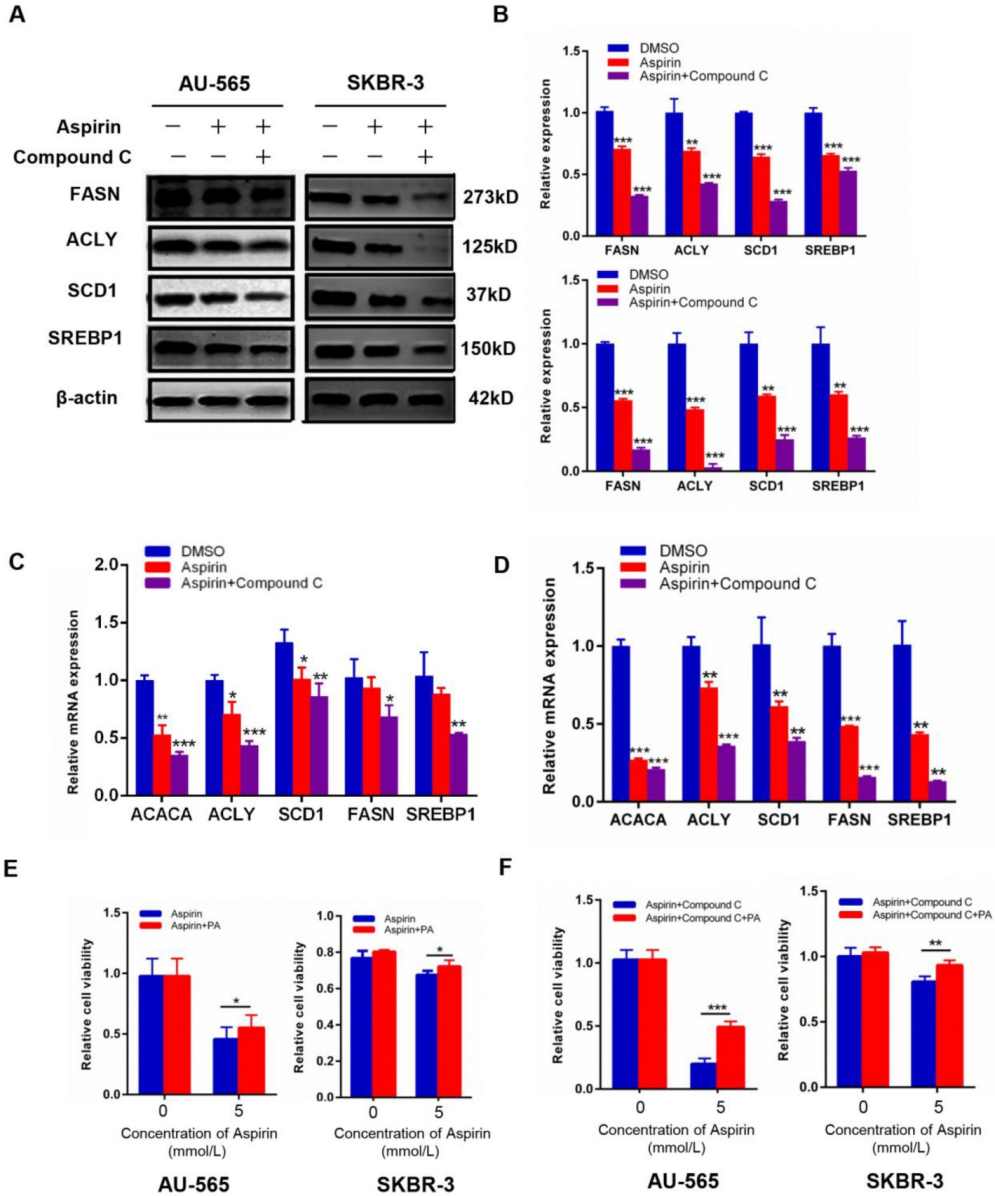
Compound C enhances aspirin-induced down-regulation of key lipid metabolism enzymes. **A-D.** Western blot (**A** and **B**) and qRT-PCR (**C** and **D**) showed that aspirin reduced the expression of SREBP1/SCD1/FASN/ACLY in AU-565 and SKBR-3 cells.** E, F.** Palmitic acid treatment reversed the decrease in cell viability caused by Compound C and aspirin in AU-565 (left) and SKBR-3 cells (right). *P < 0.05; **P < 0.01; ***P < 0.001 vs. control.

As shown in Figure 3F, lipid metabolism is a complicated process in human body, *de novo* fatty acid formation is accompanied by the activation of lipid metabolism-related enzymes. To reinforce our above results, we measured the expression of these key enzymes. We found that aspirin significantly reduced the protein and mRNA levels of SREBP1, SCD1, acetyl-CoA carboxylase alpha (ACC1), FASN, and ACLY in AU-565 and SKBR-3 cells (Figure 4A-D), which are critical enzymes for *de novo* fatty acid synthesis. The changes of these markers were also measured in MDA-MB-231 breast cancer cells (Supplementary Figure 1F). Palmitic acid is an important kind of fatty acid which can produce energy 22 and we used palmitic acid as an exogenous fatty acid to see if the supply of exogenous lipid could rescue the growth inhibition effect in our study.

AU-565 and SKBR-3 cells were treated with aspirin, with or without Compound C pre-treatment, in the presence or absence of palmitic acid. Palmitic acid rescued the decreased cell viability caused by aspirin and Compound C (Figure 4E-F). These results confirmed that aspirin inhibited HER-2-positive breast cancer cell growth in combination with Compound C by repressing* de novo* lipogenesis.

### Compound C and aspirin synergistically inhibit cancer cell* de novo* lipogenesis by regulating c-myc

**Figure 5:**
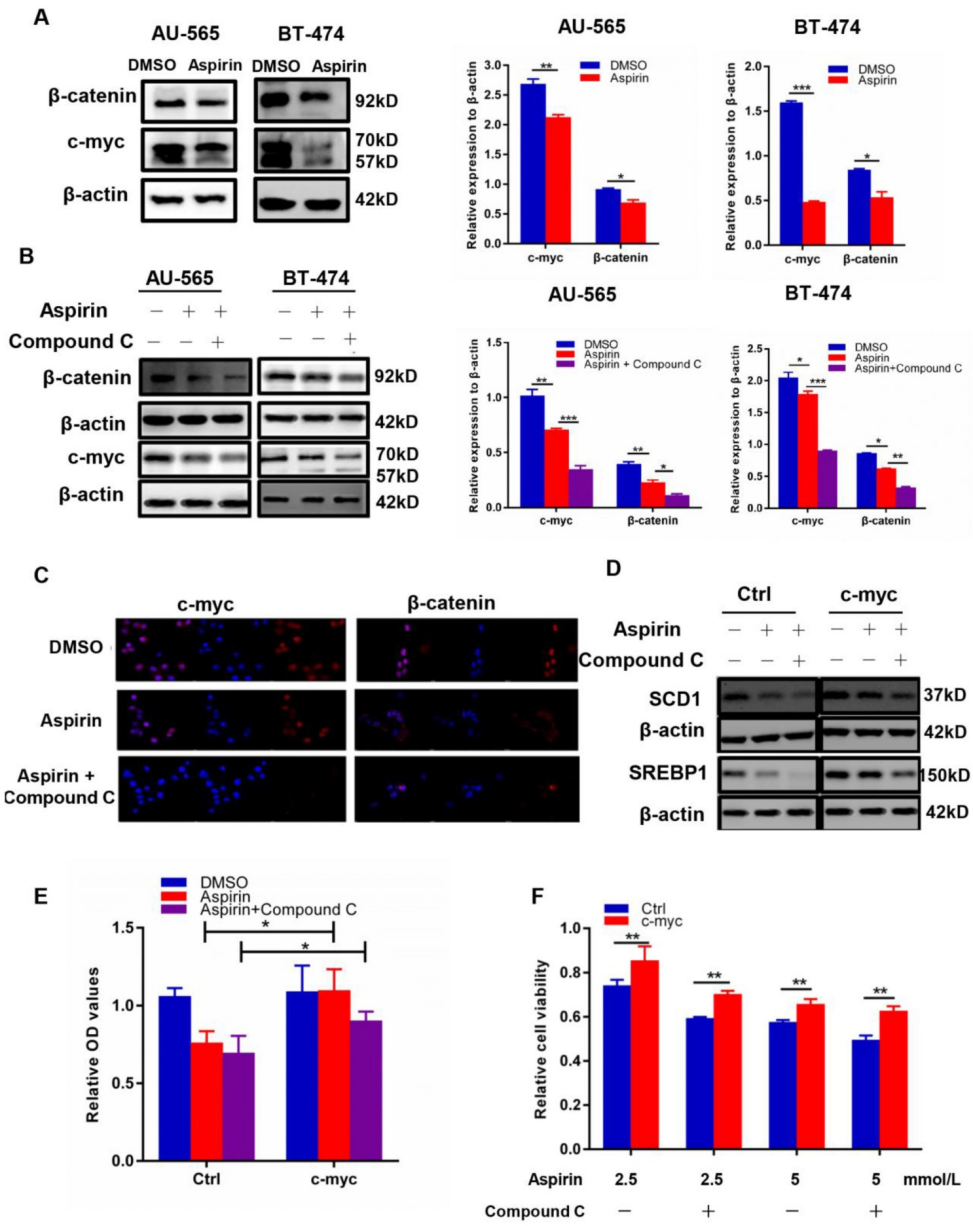
Aspirin and Compound C inhibited HER-2-positive breast cancer cell lipogenesis via c-myc regulation. **A.** Western blotting showed that aspirin inhibited c-myc and β-catenin expression in AU-565 and BT-474 breast cancer cells. **B.** Western blot showed that Compound C pre-treatment enhanced the aspirin-induced down-regulation of β-catenin and c-myc expression in AU-565 and BT-474 breast cancer cells. **C.** Immunofluorescence showed that Compound C pre-treatment strengthened the aspirin-induced down-regulation of β-catenin and c-myc in AU-565 cells. **D.** c-Myc transfection enhanced the expression of SREBP1/SCD1 in AU-565 cells. **E.** C-myc transfection enhanced the lipid formation after it was decreased in aspirin-treated AU-565 cells with or without Compound C pre-treatment. **F.** c-Myc transfection rescued cell viability impaired by aspirin with or without Compound C pre-treatment. *P < 0.05; **P < 0.01; ***P < 0.001 vs. control.

It has been reported that aspirin can regulate cancer cell proliferation by reducing the expression of c-myc, a widely known transcriptional target of β-catenin 23. Myc was able to induce lipid synthesis 24, and the c-myc/EGLN1-mediated induction of lymphoid-specific helicase (LSH) expression functioned as an oncogene and an important inhibitor of ferroptosis in carcinogenesis by promoting the expression of lipid metabolism-related genes 25. Moreover, the inhibition of β-oxidation led to lipid accumulation after MYCN inhibition 26. It was suggested that metabolic reprogramming mediated by myc during tumorigenesis was dependent on the tissue type 27. Here, we found that aspirin inhibited c-myc and β-catenin expression in AU-565 and BT-474 cells through western blotting (Figure 5A), and that Compound C intensified this effect (Figure 5B-C). We further found that c-myc transfection reversed the decreased expression of SREBP1 and SCD1 (Figure 5D) induced by aspirin and Compound C, which was sufficient to rescue lipid formation (Figure 5E) and restore the attenuated cell viability caused by aspirin and Compound C (Figure 5F). These results demonstrated that Compound C and aspirin synergistically inhibited HER-2-positive breast cancer cell growth by reducing c-myc-regulated *de novo* lipogenesis.

### Combined aspirin and Compound C treatment has a strong synergistic effect against growth of HER-2-positive breast tumors in mice

**Figure 6:**
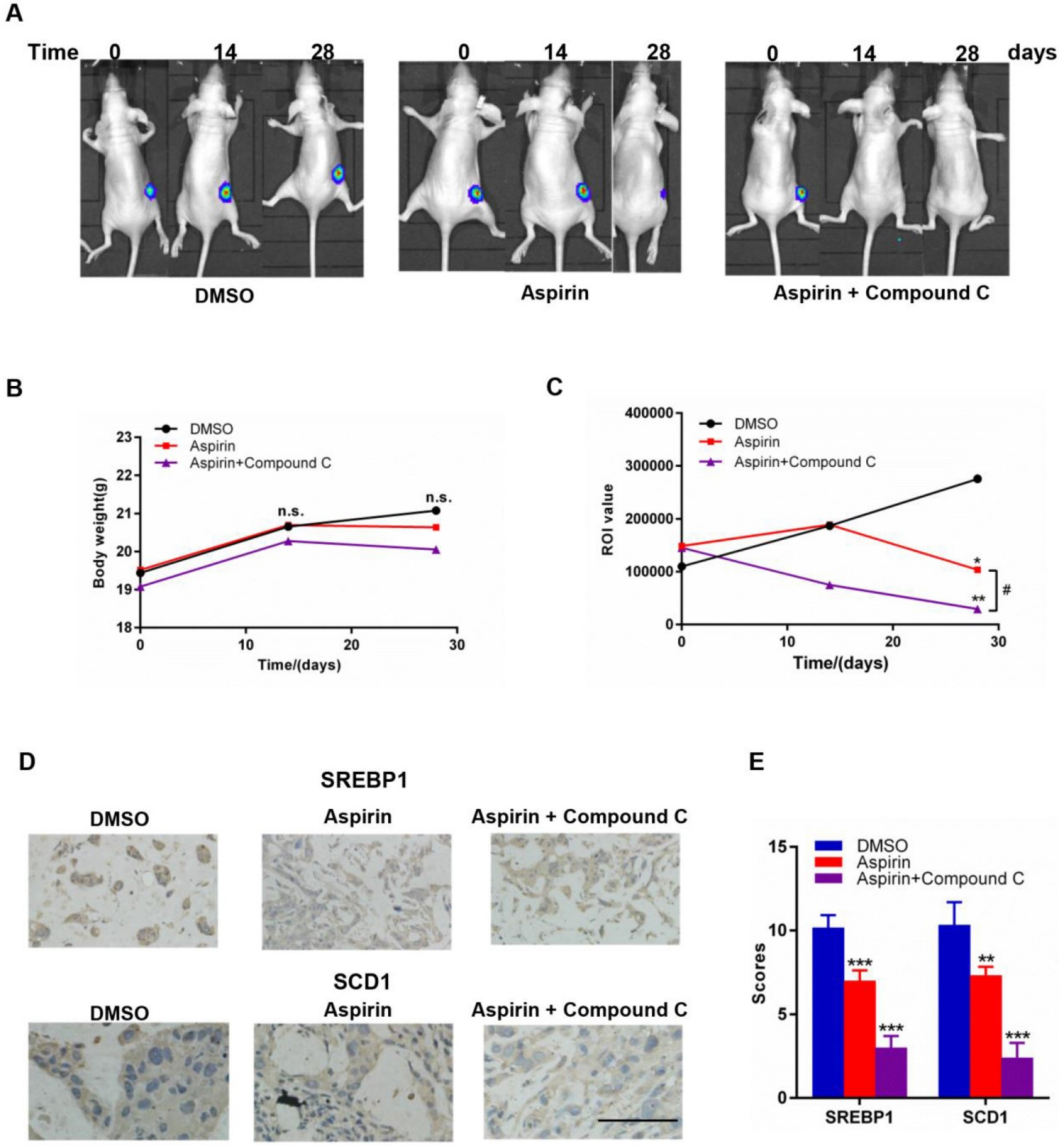
Effects of aspirin and Compound C on SKBR-3 tumor xenografts. Nude mice bearing SKBR-3-luciferase-derived tumors were treated with aspirin with or without Compound C. **A.** Representative images of nude mice in each group. **B.** Body weight of mice in each group. **C.** Aspirin and Compound C inhibited SKBR-3-luciferase-derived tumor growth. **D.** IHC was performed to investigate SREBP1 and SCD1 expression in tumor tissues. **E.** Summary of **D**. *P < 0.05; **P < 0.01; ***P < 0.001 vs. control; #*P*<0.05 between the two group. (scale bar, 100 μm).

**Figure 7:**
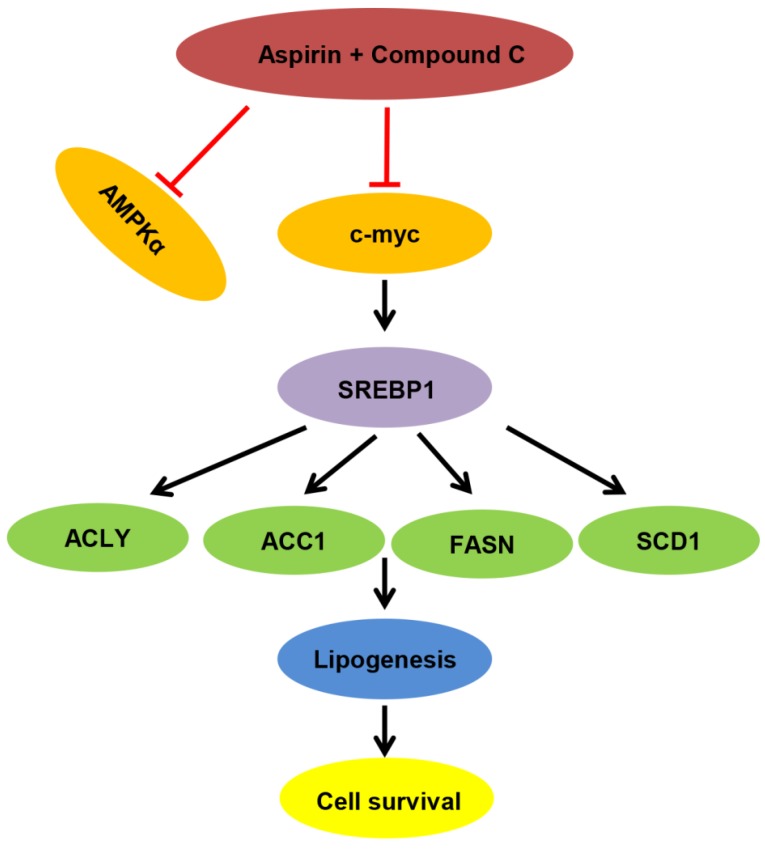
Proposed model of lipid metabolism regulation in HER-2 positive breast cancer. Aspirin and Compound C inhibit c-myc synergistically. Upon c-myc signaling inhibition, the SREBP1 and its target gene expression are downregulated sequentially, which leads to decreased lipid biosynthesis and cell growth.

To investigate the combinational effect of aspirin and Compound C on HER-2-positive breast tumor growth *in vivo*, we established a xenograft model in female mice by injecting them with stably overexpressing SKBR-3-luciferase-positive cells; mice were then treated with aspirin with or without Compound C for 30 days. As shown in Figure 6A and 6C, the return on investment (ROI) values of tumors in both of the two experimental groups were significantly lower than those in the control group, and those in the combined treatment group were significantly lower than those in the aspirin-treated group (Figure 6C); however, the body weight within each group showed no significant difference (Figure 6B), suggesting that the combination of aspirin and Compound C dramatically suppressed the growth of HER-2-positive breast tumors without affecting the overall wellbeing of the mice and Compound C enhanced the antitumor effect of aspirin *in vivo*. We next measured SREBP1 and SCD1 protein expression in xenograft tumor tissues. Compared to the control group, the expression of SREBP1 and SCD1 was significantly decreased in aspirin-treated group, and combined treatment with Compound C could further decreased their expression (Figure 6D-E); this was consistent with the results of our *in vitro* experiments. Overall, these findings suggested that combined aspirin and Compound C treatment inhibited HER-2-positive breast tumor growth, possibly through the inhibition of key lipogenesis-related enzymes in HER-2-positive breast tumors (Figure 7).

## Discussion

HER-2-positive breast cancer is a highly aggressive subtype of breast cancer. The number of HER-2 proteins in HER-2-positive cancer cells is approximately 2 × 10^6^; this is about 100 times more than normal cells 2, which allow cancer cells to proliferate more rapidly. Thus, treating HER-2-positive breast cancer is quite difficult. Although great efforts have been made in the treatment and management of HER-2-positive breast cancer, the discovery of new drugs is still of major importance.

Aspirin has attracted the attention of many investigators in terms of its role in cancer prevention in recent decades. Many studies have indicated that aspirin shows protective effects mainly against colorectal, esophageal, gastric, and endometrial cancers 28, 29. Several clinical studies also showed that aspirin intake may decrease the risk of *in situ* breast tumors or hormone receptor-positive tumors, rather than affecting the overall risk of breast cancer 30. However, the association between aspirin treatment and HER-2-positive breast cancer has not been studied in detail. Nath et al. proved that aspirin inhibited HER-2-positive breast cancer SKBR-3 cell growth by regulating nuclear factor κB (NF-κB) activity 31. Our study indicated that aspirin exerted an anticancer activity in the HER-2-positive breast cancer cell lines AU-565, BT-474 and SKBR-3 by inhibiting their proliferation and colony-forming abilities, regulating cell cycle progression, and inducing apoptosis, through the activation of AMPK signaling.

AMPK has been implicated in a range of biological functions including cell polarity, autophagy, apoptosis, and cell migration 32. Compound C, a widely known AMPK inhibitor 33, was used to reverse the aspirin-induced activation of AMPK. Unexpectedly, we found that Compound C pre-treatment, after successfully blocking AMPK activation, enhanced the growth inhibition effect induced by aspirin on HER-2-positive breast cancer cells, but could not affect the anti-growth effect of aspirin in HER-2 negative breast cancer cells. In the Compound C pre-treated group, we observed more robust inhibition of cell proliferation, a reduced colony-forming ability, and a higher apoptosis rate than the aspirin alone-treated HER-2-positive breast cancer cells. The increased inhibition of HER-2-positive breast cancer cell biological function upon Compound C pre-treatment was surprising; thus, we began to study and analyze the mechanisms surrounding this. Interestingly, Compound C, despite being an AMPK inhibitor, can inhibit cell growth in 3T3-L1 preadipocytes 34 and vascular smooth muscle cells 35 independent of AMPK activity. Compound C is also known to enhance tumor necrosis factor (TNF)-related apoptosis-inducing ligand (TRAIL)-induced apoptosis through a caspase-dependent mechanism in Caki cells, independent of AMPK 33. The previously reported Compound C-induced inhibition of macrophage migration was shown to be mediated via the inhibition of the focal adhesion kinase, AKT, and NF-κB pathways, but was independent of AMPK activation 36.

In light of these reported findings, we attempted to uncover the differences in signaling after each treatment. Fatty acids are the main substrates for energy production and membrane biogenesis; the shift in the lipid acquisition mechanism from lipid uptake toward *de novo* lipogenesis dramatically changes the membrane properties and protects cells from both endogenous and exogenous injury, thus maintaining tumorigenesis 37.

Our findings that aspirin can reduce lipogenesis, as determined by LC-MS/MS and Oil red O staining, were consistent with those of a previous clinical trial 38, which showed a reduction in cholesterol, triglyceride, and free fatty acid production after aspirin treatment in a mouse model of adiposity. In HER-2-positive breast cancer cells, Compound C pre-treatment made the attenuation of lipogenesis in the aspirin-treated group more pronounced. Furthermore, our data suggested that the addition of palmitic acid could successfully rescue the impaired cell viability in aspirin and Compound C-treated cells. Hence, our study clearly demonstrates that aspirin, in combination with Compound C, inhibits HER-2-positive breast cancer cell growth by reducing fatty acid synthesis.

We found that aspirin deregulated the expression of β-catenin and c-myc. It has been reported that in tumor cells, c-myc plays an important role in tumorigenesis 39, and that c-myc activity is required for normal cell proliferation 32. Therefore, the activation of intrinsic tumor suppression can be triggered only when c-myc signaling is oncogenic 32. Subtle changes in c-myc expression and its dysregulation can have a profound effect on tumorigenesis in animal models 40. Our study showed that combined Compound C and aspirin treatment induced the increased down-regulation of β-catenin and c-myc expression, independent of AMPK activity.

Several lines of evidence have confirmed that myc induces either fatty acid synthesis or oxidation, and studies have shown that aspirin can also deregulate cancer cell lipid metabolism; this was confirmed in the present study.

Next, we researched the relationship between c-myc expression and aspirin-mediated lipogenesis inhibition. As expected, we found that aspirin and Compound C down-regulated lipogenesis by targeting c-myc in HER-2-positive breast cancer cells. It was confirmed c-myc could rescue the down-regulated expression of SREBP1 and SCD1 mediated by aspirin and Compound C treatment, as well as enhance lipid formation and cell viability to some extent.

Furthermore, we constructed a HER-2-positive breast cancer xenograft tumor model in female mice; consistent with the effects observed *in vitro*, we found that mice treated with Compound C and aspirin showed tumors with a smaller size and lower expression of SREBP1 and SCD1 compared to those treated with aspirin monotherapy without affecting the overall wellbeing of the mice in all groups.

In summary, we demonstrated that aspirin inhibited HER-2-positive breast cancer cell viability in combination with Compound C through an AMPK-independent mechanism. Previous clinical trials demonstrated that aspirin can regulate lipid metabolism 41, and more recently, studies have linked fatty acid synthesis with oncogenic myc activity 42. As summarized in Figure 7, our present findings demonstrate for the first time that aspirin and Compound C inhibited expression of lipogenesis-related key enzymes via c-myc inhibition to attenuate fatty acid metabolism and inhibit tumor growth in HER-2-positive breast cancer. Therefore, although further investigations are required to determine the underlying mechanisms, our study shows the potential for combined aspirin and Compound C treatment as a pharmacological tool for the prevention and treatment of HER-2-positive breast cancer.

## Supplementary Material

Supplementary figure S1.

## Abbreviations

HER-2: Human Epidermal Growth Factor Receptor 2
AMPK: AMP-activated protein kinase
COX: cyclooxygenase
PG: prostaglandin
TXA2: thromboxane A2
SREBPs: Sterol-regulatory element binding proteins
SCD1: Stearoyl-CoA desaturase-1
FASN: Fatty acid synthase
ACLY: ATP citrate lyase
FBS: fetal bovine serum
MTT: 3-(4,5-dimethylthiazol-2-yl)-2, 5-diphenyltetrazolium bromide
PBS: phosphate-buffered saline
PI: propidium iodide
EDTA: ethylenediaminetetraacetic acid
PAGE: polyacrylamide gel electrophoresis
PVDF: polyvinylidene fluoride
TBST: Tris-buffered saline (TBS)/Tween 20
MS: mass spectrum
HRP: horseradish peroxidase
IHC: Immunohistochemistry
LD: Lipid droplets
MUFAs: Monounsaturated fatty acids
