# Supplementary material for: Compound C enhances the anticancer effect of aspirin in HER-2-positive breast cancer by regulating lipid metabolism in an AMPK-independent pathway
Source: Int J Biol Sci. 2020 Jan 1;16(4):583–97. doi: 10.7150/ijbs.39936 (PMC6990926; doi:10.7150/ijbs.39936)
Supplement: Supplementary file 1 — Supplementary figure S1. [file ijbsv16p0583s1.pdf]

Supplementary Figure1

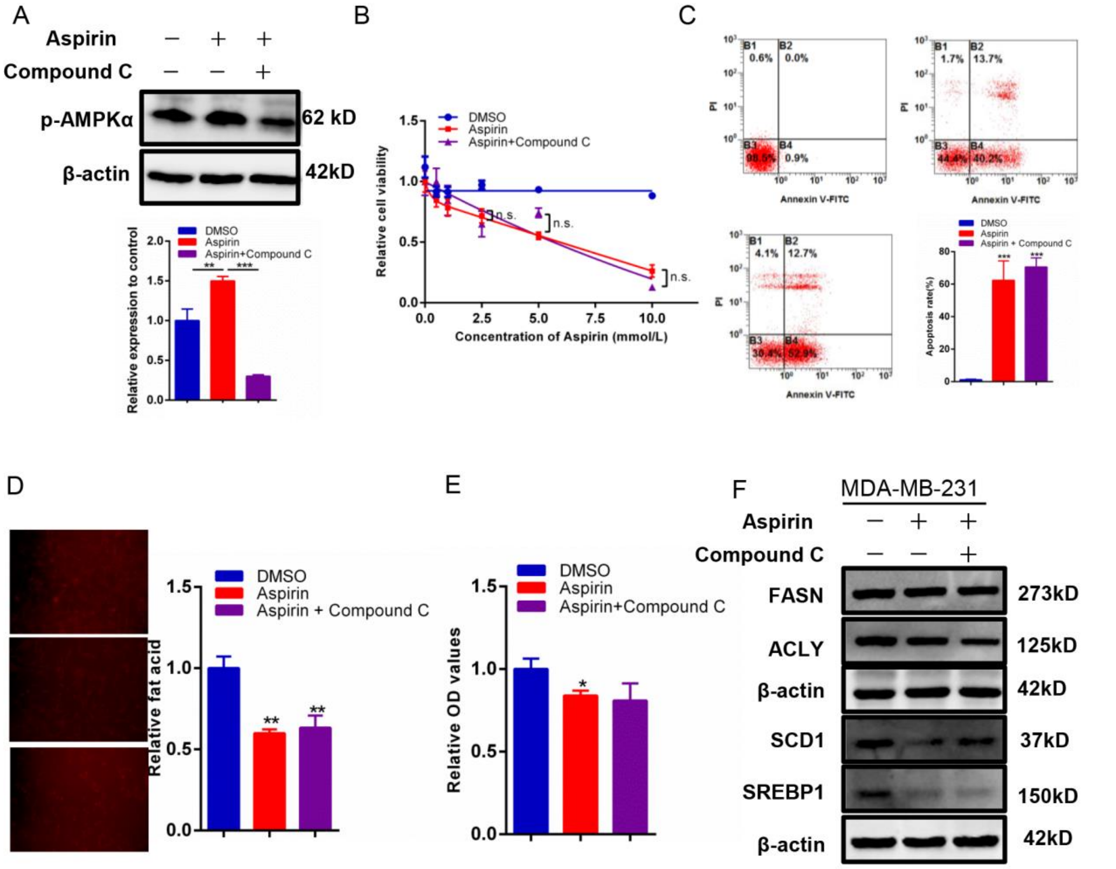

A. Compound C successfully inhibited the expression of p-AMPK elevated by aspirin in MDA-MB-231 cells. **B**. An MTT assay showed that Compound C (10 μmol/L, 4 h), did not affect the anti-growth effect of aspirin in MDA-MB-231 cells. **C**. Flow cytometry showed that aspirin induced apoptosis in MDA-MB-231 breast cancer cells, without being affected by Compound C. **D. E.** An Oil red O staining assay showed that aspirin impaired lipogenesis in MDA-MB-231 breast cancer cells, without being exacerbated by Compound C. **F**. Western blot showed that aspirin reduced the expression of SREBP1/SCD1/ACLY in MDA-MB-231 cells. $*P < 0.05$; $**P < 0.01$; $***P < 0.001$ vs. control.
